# Supplementary material for: Yiai Fuzheng decoction inhibits triple-negative breast cancer by remodeling the immune microenvironment
Source: Front Immunol. 2025 Sep 30;16:1615631. doi: 10.3389/fimmu.2025.1615631 (PMC12518410; doi:10.3389/fimmu.2025.1615631)
Supplement: Supplementary file 2 [file DataSheet1.zip › Supplementary File 2/Treatment/D_vs_H/07_Pathway_Analysis/Pathway_Analysis/Sig_Pathways_ID/Citrate cycle (TCA cycle).pdf]

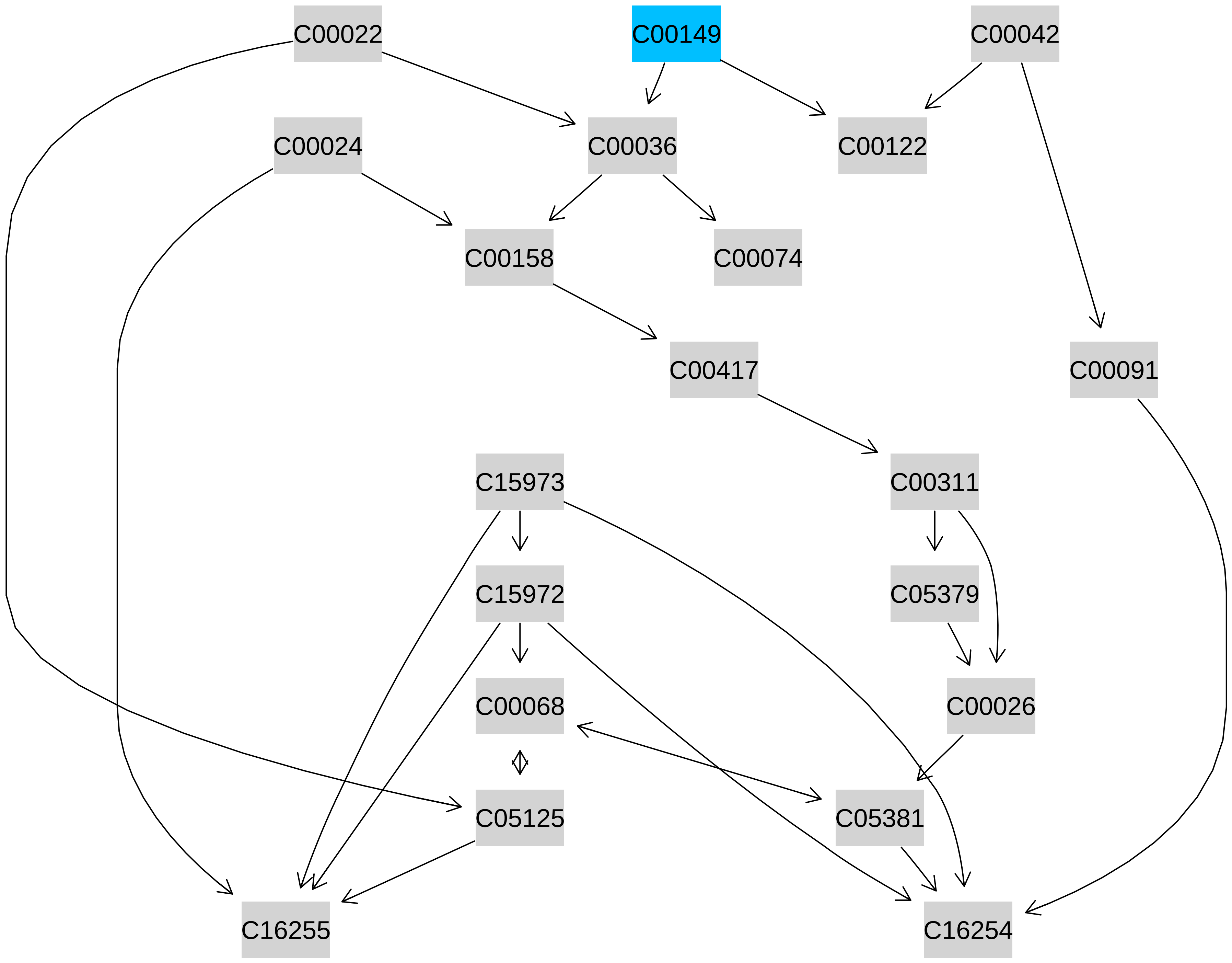
C00022
C00149
C00042
C00024
C00036
C00122
C00158
C00074
C00417
C00091
C15973
C00311
C15972
C05379
C00068
C00026
C05125
C05381
C16255
C16254
